# Current role of colonoscopy in infants and young children: a multicenter study

**DOI:** 10.1186/s12876-019-1060-7

**Published:** 2019-08-20

**Authors:** Ryusuke Nambu, Shin-ichiro Hagiwara, Fumihiko Kakuta, Tomoko Hara, Hirotaka Shimizu, Daiki Abukawa, Itaru Iwama, Seiichi Kagimoto, Katsuhiro Arai

**Affiliations:** 1 Division of Gastroenterology and Hepatology, Saitama Children’s Medical Center, 1-2 Shintoshin, Chuo-ku, Saitama-city, Saitama, 330-8777 Japan; 2 Division of General Pediatrics, Saitama Children’s Medical Center, Saitama, Japan; 3 Division of General Pediatrics and Gastroenterology, Miyagi Children’s Hospital, Miyagi, Japan; 4 Division of Gastroenterology, National Center for Child Health and Development, Tokyo, Japan

**Keywords:** Colonoscopy, Infants and young children, Eosinophilic gastrointestinal disorders, Inflammatory bowel diseases, Monogenic disease

## Abstract

**Background:**

To evaluate the role of colonoscopy in infants and young children and clarify the distribution of colonoscopy-requiring diseases in this age group.

**Methods:**

Cohorts of colonoscopies performed at three children’s hospitals in Japan between April 2011 and March 2016 including infants and children younger than six years of age were retrospectively reviewed.

**Results:**

In total, 453 colonoscopies were performed in 276 infants and young children. Of these 275 (60.8%) were for diagnostic purposes, 177 (39.2%) were performed as follow-up, and one case was performed for treatment. The median patient age at the time of diagnostic colonoscopy was 2.49 years, and there was a male-to-female ratio of 1.72:1. Abnormal macroscopic and/or histopathological findings were noted in 212 (77.1%) cases. Of these, definite diagnoses were established for the presence of eosinophilic gastrointestinal disorders (EGIDs), inflammatory bowel disease (IBD), and polyp/polyposis in 23, 18.5, and 14% of patients, respectively. Among 51 IBD cases, ulcerative colitis, Crohn’s disease, and IBD-unclassified were identified in 47.1, 33.3, and 7.8%, retrospectively via endoscopic examination. Of these, 11 (22%) were eventually diagnosed with monogenic diseases via genetic testing. Of those with rectal bleeding, EGIDs, polyps/polyposis, and IBD were found in 27, 19, and 18%, retrospectively. There were significantly more cases of EGIDs and fewer ones of IBD and polyps/polyposis in patients with rectal bleeding younger than two years of age. Furthermore, 68% of all follow-up colonoscopies were performed in children with IBD. There were no serious complications in our study cohort.

**Conclusion:**

We determined the role of colonoscopy in infants and young children. Diseases diagnosed using colonoscopy in this age group included IBD, EGIDs, and polyps/polyposis. The increasing trend of patients with IBD and EGIDs worldwide means that the role of colonoscopy in infants and younger children will be more important in the future.

## Background

Advances in anesthetic techniques and the evolution in the size and flexibility of endoscopes, have led to an increase in the number of colonoscopies performed in children worldwide, including infants and young children [1]. Although the major role of colonoscopy is the screening and diagnosis of colon cancer in adults [2], there are few children who are diagnosed with colon cancer. Frequently diagnosed diseases during childhood include inflammatory bowel diseases (IBD), polyps/polyposis, and graft versus host disease; however, there have been few reports demonstrating the role of colonoscopy in infants and young children [3, 4].

A large multicenter study conducted in the United States reported that most prevalent indications for pediatric colonoscopy include rectal bleeding (31%), abdominal pain (31%), and diarrhea (24%) [5]. Epidemiological studies have revealed a rise in the incidence of IBD and eosinophilic gastrointestinal disorders (EGIDs) in children including those younger than six years of age. IBD found in this age group may indicate IBD associated with primary immunodeficiency [6–8]. Bequet et al., reported that the most common phenotype found in very early onset IBD was colitis [9], which frequently presents with rectal bleeding.

Over the past decades, the role of colonoscopies in infants and young children has not been well described. The present study aimed to elucidate the role of colonoscopy and the diagnosis requiring for colonoscopy in infants and young children.

## Methods

We conducted a retrospective analysis of a cohort of children who underwent colonoscopies at the following three tertiary children’s hospitals in Japan; the National Center for Child Health and Development, Tokyo; Miyagi Children’s Hospital, Miyagi; and Saitama Children’s Medical Center, Saitama. Each institution had at least two pediatric gastroenterologists certified by the Japanese Society for Pediatric Gastroenterology, Hepatology and Nutrition.

A total of 1417 colonoscopies were performed in children aged 18 years old or younger between April 2011 and March 2016 at the aforementioned institutions. The following data were collected from the electronic databases of each hospital: age, sex, type of anesthesia, extent of colonoscopy, primary indication, abnormal macroscopic and/or histopathological findings, definite diagnosis, and serious complications. Diagnostic colonoscopy, follow-up colonoscopy, and other reasons for colonoscopy were also distinguished. Patients younger than six years of age were defined as “infants and young children”. Eosinophilic gastrointestinal disorders (EGIDs) were defined as disorders that primarily affect the gastrointestinal tract with eosinophil-rich inflammation in the absence of other known causes for eosinophilia, such as a drug reaction, parasitic infection, and malignancy [10].

### Statistical analyses

All data were summarized and present as mean ± standard deviation for continuous variables. We considered values < 0.05 were considered statistically significant. We performed all statistical analyses using EZR, a graphical user interface for R (The R Foundation for Statistical Computing) that is a specifically modified version of R Commander designed to add statistical functions frequently used in biostatistics [11].

### Ethics statement

Our study protocol was approved by the human ethics committees of the National Center for Child Health and Development (1460), Miyagi Children’s Hospital (MiyaKoRinriDai327); and Saitama Children’s Medical Center (2016–06-006). Patient information was anonymously handled at each institution, and the opportunity to withdraw from participation was provided to all participants and their guardians by posting flyers around the hospitals.

## Results

**Fig. 1:**
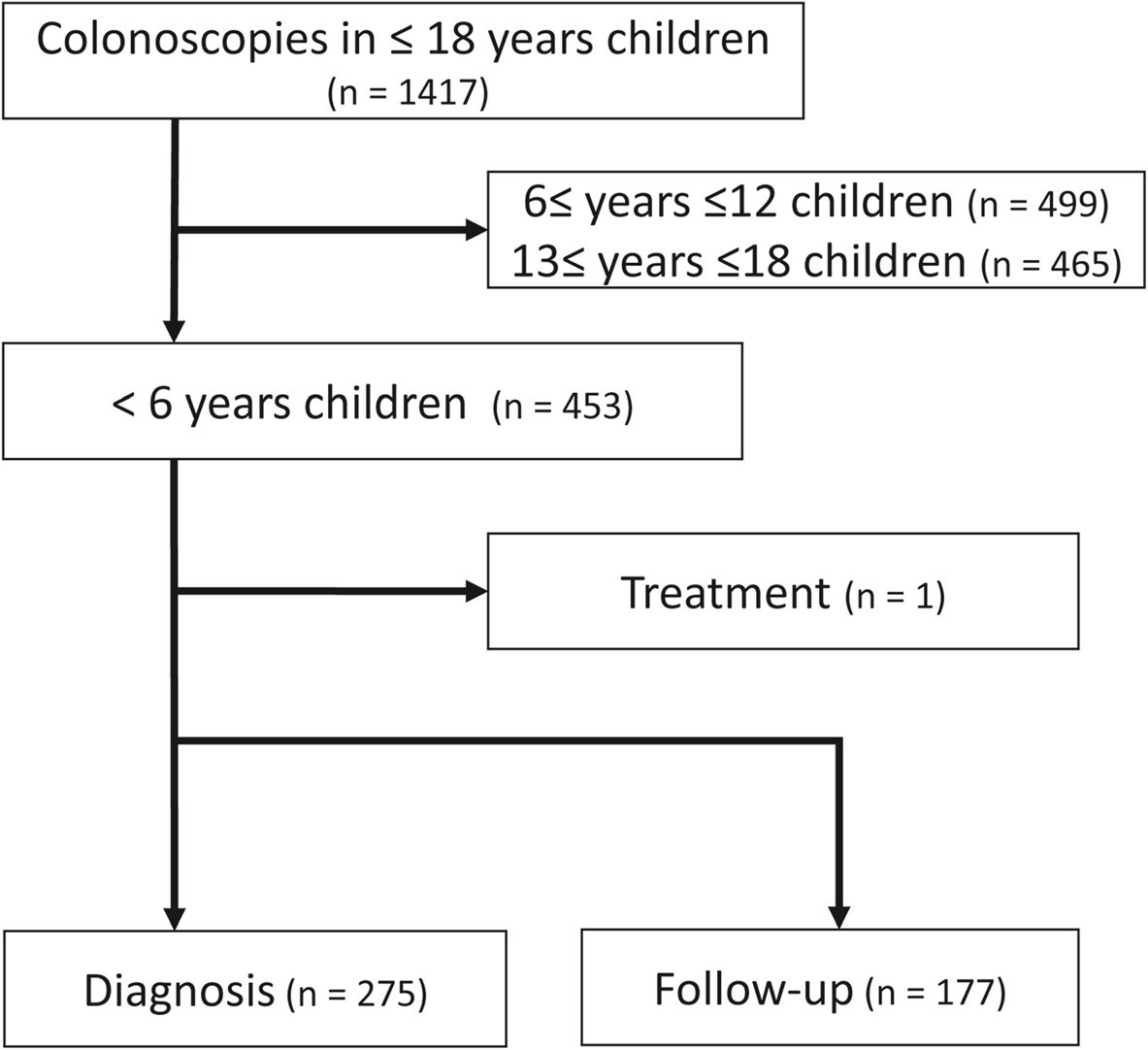
Purpose for colonoscopy in infants and young children in the present study. Colonoscopies were performed in patients aged ≤18 years between April 2011 and March 2016 at three tertiary centers

Of the 1417 colonoscopies performed at the three children’s hospitals, 453 procedures (32.0%) were performed in infants and young children. Of these 453 colonoscopies performed in infants and young children, 275 (60.8%) were performed as “diagnostic procedure” and 177 (39.2%) were “follow-up procedure” (Fig. 1). One colonoscopy was conducted for the decompression of a mechanical ileus secondary to colonic stenosis, which was complicated with necrotizing enterocolitis.

Of the 275 diagnostic colonoscopies, the median age at the time of colonoscopy was 2.49 years (range: 5 days - 5 years 11 months) with a male-to-female ratio of 1.72:1 (Table 1). One hundred twenty-three procedures (44.7%) were performed in children less than two years old. Additionally, 130 (47%), 124 (45%) and 22 (8.0%) procedures were performed with general anesthesia, intravenous sedation, and no sedation, respectively. Nineteen of 22 procedures performed with no sedation were done in infants (<12mo), while the remaining three cases were confined to the sigmoid colon. Ileoscopy was performed in 170 (62%) of the 275 diagnostic colonoscopies. In 111 (85%) cases of the 130 colonoscopies with general anesthesia, terminal ileum were reached. Of note this rate was much higher than that observed in the colonoscopy group with sedation (54/124 procedures. 44%). Two hundred twelve procedures (77.1%) showed abnormal macroscopic and/or histopathological findings. There were 63 cases of EGIDs (23%), 51 cases of IBD (18.5%), 38 cases of polyps/polyposis (14%), 37 cases of nonspecific colitis (13.5%), 13 cases of hemorrhoid/anal fissure (4.7%), five cases of lymphangiectasis (1.8%), and four with IgA vasculitis (1.5%) (Table 2). These definite diagnoses were made from endoscopic and histological findings coupled with the representative patients’ with clinical courses. Among the 51 patients with IBD, 24(47.1%) were endoscopically diagnosed with ulcerative colitis (UC), 18(33.3%) with Crohn’s disease (CD), one with intestinal Behçet disease, and remaining four were diagnosed as IBD-unclassified (IBD-U), respectively. Eleven (22%) children with IBD were also eventually diagnosed via genetic testing with monogenic diseases, including chronic granulomatous disease (*n* = 6), familial Mediterranean fever (*n* = 1), Wiskott-Aldrich syndrome (*n* = 1), STAT-3 gain of function (*n* = 1), Hoyeraal Hreidarsson syndrome (*n* = 1), and activated PI3K-delta syndrome (*n* = 1). Fifty-three percent of children younger than two years of age with IBD were later diagnosed with monogenic diseases associated with IBD.

**Table 1:** Clinical character and disease distribution of diagnostic colonoscopies

	*n* = 275
Gender, male: female	174:101
Age, yr., median ± SD	2.49 ± 1.47
age < 2 yr	123 (45%)
2 yr. ≤ age < 6 yr	152 (55%)
*Type of anesthesia*	
general	130 (47%)
intravenous	124 (45%)
no sedation	22 (8.0%)
*Extent of colonoscopy*	
~ ileum	170 (62%)
~ cecum	8 (2.9%)
~ ascending colon	9 (3.3%)
~ transverse colon	28 (10.2%)
~ descending colon	33 (12%)
~ sigmoid colon	27 (9.8%)
Abnormal findings	212 (77%)
*Primary indication*	
Rectal bleeding	206 (75%)
Diarrhea	36 (13%)
Abdominal pain	6 (2.2%)
Fail to thrive	4 (1.5%)
Repetitive intussusception	4 (1.5%)
Anal fistula	3 (1.1%)
Anemia	3 (1.1%)
Hypoalbuminemia	3 (1.1%)
Others*	10 (3.6%)

* “Others” refers to diseases identified in fewer than two children

**Table 2:** Disease distribution of diagnostic colonoscopies

	*n* = 275
EGIDs	63 (23%)
IBD	51 (19%)
Normal	41 (15%)
Polyps/Polyposis	38 (14%)
Nonspecific Colitis	36 (13%)
Hemorrhoid/MPS	13 (4.7%)
Lymphangiectasis	5 (1.8%)
IgA vasculitis	4 (1.5%)
Others*	24 (8.7%)
Normal	41 (15%)

*EGIDs* eosinophilic gastrointestinal disorders (including gastrointestinal allergy), *IBD* inflammatory bowel diseases, *MPS* mucosal prolapse syndrome

* “Others” refers to diseases identified in fewer than two children

In all diagnostic colonoscopies, rectal bleeding was the most common primary indication (*n* = 206; 75%) (Table 1). Diagnoses found among the infants and young children showing rectal bleeding included EGIDs (*n* = 52; 25%), polyps/polyposis (*n* = 37, 18%), and IBD (*n* = 36, 17%). We compared the nature of disease distribution in children younger than two years old with that in children with two to five years old. In the former, the most prevalent diagnosis was EGIDs, and the frequency rate of such was higher in comparison with in those aged two-five years old (43% vs. 12%; *P < 0.001*) (Table 3). Conversely, polyps/polyposis and IBD tended to be found more frequently in the older group (polyps/polyposis: 6.8% vs. 26%; *P < 0.001,* IBD: 10.8% vs. 23.3%; *P = 0.06*).

**Table 3:** Cause of rectal bleeding required by age group

	0 ≤ yr. < 2	2 ≤ yr. < 6	*P-*value
	(*n* = 88)	(*n* = 118)	
Gender, male: female	51:37:00	75:43:00	
Age, yr., median ± SD	0.82 ± 0.54	3.83 ± 1.09	
*Diagnosis*			
EGIDs	38 (43%)	14 (12%)	< 0.001
Polyps/Polyposis	6 (6.8%)	31 (26%)	< 0.001
IBD	10 (11%)	26 (22%)	0.06
Nonspecific Colitis	20 (23%)	14 (12%)	0.05
Hemorrhoid/MPS	1 (1.1%)	11 (9.3%)	0.01
Others*	6 (6.8%)	9 (7.6%)	
Normal	7 (8.0%)	13 (11%)	

*EGIDs* eosinophilic gastrointestinal disorders (including gastrointestinal allergy), *IBD* inflammatory bowel diseases, *MPS* mucosal prolapse syndrome

* “Others” refers to diseases identified in fewer than two children

A total of 177 colonoscopies were performed as follow-up procedures. Of these, 68% of procedures were performed in patients with IBD (Table 4). In these “follow-up” colonoscopies, six children who were eventually diagnosed with monogenic diseases underwent a total of 29 colonoscopies (4.8 colonoscopies per patient). Similarly, 16 children with UC underwent 52 colonoscopies (3.2 colonoscopies per patient) and 10 children with CD underwent 22 colonoscopies (2.2 colonoscopies per patient). No serious complications such as perforations or severe bleeding occurred.

**Table 4:** Disease distribution in follow up colonoscopies

Follow-up	*n* = 177
IBD	120 (68%)
EGIDs	15 (8.5%)
Nonspecific Colitis	14 (7.9%)
Polyps/Polyposis	13 (7.3%)
Others*	15 (8.5%)

*EGIDs* eosinophilic gastrointestinal disorders (including gastrointestinal allergy), *IBD* inflammatory bowel diseases

* “Others” refers to diseases identified in fewer than two children

## Discussion

As part of the present research, we retrospectively reviewed 453 colonoscopies performed in infants and young children younger than six years of age at three tertiary care children’s hospitals in Japan. To the best of our knowledge, this is the first study to evaluate the role of colonoscopy in infants and young children. The proportion of colonoscopies performed in infants and young children was 32.0% of the 1417 colonoscopies recorded in children aged 18 years old or younger. A cursory review of the literature revealed that this rate was higher than those in other previous studies on the subject of pediatric colonoscopy [12, 13]. We suggest two reasons for this findings; first, our three institutions are the tertiary children’s hospitals having pediatric anesthesia, and second, colonoscopies in children aged 15 to 18 years old in Japan are most often performed by gastroenterologists in adult units. The terminal ileum was reached in 62% of the diagnostic colonoscopies reviewed in this study. Reasons for incomplete colonoscopy include severe colitis with a high risk, cases not indicated for completion by the senior endoscopist, poor bowel preparation, and technical failure. Approximately 75% of the diagnostic colonoscopies revealed abnormal macroscopic and/or histopathological findings. EGIDs and IBD were the most common diagnoses, and rectal bleeding was the most common primary indication for colonoscopy in this population. Approximately 70% of follow-up colonoscopies were performed in IBD patients.

The prevalence of positive macroscopic and/or histopathological findings in diagnostic colonoscopies in our study among infants and young children was compared with the details of other studies on children either younger than 16 years or 18 years old. In Asia, the prevalence rates of positive diagnostic findings were 45.8, 50.6, and 70.5% of studies from Korea, Hong Kong and South China, respectively [13–15]. In the United Kingdom, 62% of colonoscopies performed in children younger than 16 years old had abnormal findings [16]. Lissy et al. reported that diagnostic colonoscopy revealed abnormalities in 80% of children referred with rectal bleeding [17]. In practice, negative findings are often as important as positive findings in the management of children with gastrointestinal symptoms. Our study demonstrated that colonoscopies are equally or more important in infants and young children as compared with children of other ages.

The most common diagnoses in children who underwent colonoscopy in this study were, in order, EGIDs and IBD. While a diagnosis for EGIDs requires pathological eosinophil infiltration, macroscopic and microscopic findings are nonspecific [18]. This includes gastrointestinal allergies, such as cow’s milk protein-induced enteropathy, food-protein-induced enterocolitis, proctocolitis, and allergic eosinophilic gastroenteritis. In Japan, the incidence of EGIDs in neonates and infants has been increasing since the late 1990s [19]. Our three tertiary institutions do not perform colonoscopies in all patients with suspected gastrointestinal allergies. Children with EGIDs do not require colonoscopy for diagnosis because food avoidance and oral food challenges are often sufficient to determine a diagnosis. On the other hand, as EGIDs share common clinical features with IBD, especially in the acute phase, the making of an early diagnosis using colonoscopy is important to ensure the proper management of this vulnerable population [20]. In the present study, the fact that EGIDs and IBD were the most common diagnoses found for children who underwent colonoscopy seems logical. An increase in the number of infants and young children with EGIDs and IBD in Japan would increase the demand for colonoscopies.

As indicated above, IBD was the second leading diagnosis for infants and young children included in this study who underwent colonoscopy. A recent study suggested that the incidence of IBD is increasing in young children aged four years or younger, as well as those aged between five and nine years old [8]. Since infants and young children with IBD frequently have colonic lesions, colonoscopy is important tool when IBD is suspected [9]. The UC-to-CD ratio in this study was 1.3, and this ratio differs from those in Western countries. Griffiths et al. reported that, the occurrence of pediatric UC in Scandinavian countries exceeds that of CD, whereas in both North America and in the United Kingdom, the incidence of pediatric CD was greater than that of UC [21].

Recent advances in diagnostic tools, particularly in the field of genetics, have placed a spotlight on the existence of primary immunodeficiency associated gastrointestinal disorders such as monogenic diseases among IBD patients, especially in those diagnosed with IBD before the age of six years. Atypical endoscopic and histological findings of IBD in this age group always challenge pediatric gastroenterologists, and some studies have classified this particular group of IBD cases as displaying UC-like or CD-like disease. Patients with monogenic diseases may suffer from serious infections due to the use of immunosuppressive drugs to treat IBD. On the other hand, hematopoietic stem cell transplantation may cure some of the known monogenic diseases such as interleukin 10 abnormalities, X-linked lymphoproliferative disorder type 2, or chronic granulomatous disease. It is becoming increasingly critical to suspect the monogenic IBD when making the diagnosis of IBD in infants and young children. In the present study, 22% of patients diagnosed by the age of six years and 53% diagnosed by the age of two years were later diagnosed with monogenic diseases. The overall incidence and prevalence rates of monogenic diseases remain unknown. In Japan, Suzuki et al. reported monogenic mutations in five out of 35 children younger than 16 years old with IBD (14.3%) [22]. In the United Kingdom, Kammermeier et al. reported that 19 cases (31%) of monogenic diseases were diagnosed via the genetic screening of 62 IBD patients by the age of two years [23]. Determining who to test for monogenic diseases and how to interpret the genetic test results could cause variations in the detection rate of IBD; therefore standardized methods to diagnose monogenic diseases need to be established and validated.

Follow-up colonoscopies were performed in 68% of children diagnosed with IBD. Interestingly, more colonoscopies were performed per patients in those with monogenic diseases in comparison with in cases of CD and UC. This may reflect the difficulties encountered in determining a definite diagnosis and proper management in this age group as well as the longer follow-up periods that are indicated before the age of six years, since monogenic diseases are more prevalent in children younger than two years old.

Our results demonstrate that 75% of children who underwent colonoscopy had rectal bleeding as a primary indication at diagnosis. We similarly found that rectal bleeding was the most common presentation for colonoscopies in all aged children in other recent reports [12, 15]. In this study, we compared the disease distribution in children younger than two years old with that in children with aged to two to five years old with reference to the pediatric Paris classification for IBD, because the number of IBD children has sharply increased in Japan. In the Paris classification, infantile (and toddler) onset IBD (disease onset in those younger than two years old) was highlighted because of the suggestions of an increased genetic component, a severe disease course, and of being at a particularly high risk for an underlying primary immunodeficiency [24]. Polyps/polyposis and IBD tended to be more frequently found in those aged 2–5 years in this study than in those aged less than two years, whereas EGIDs were more frequent in children younger than two years than in those aged 2–5 years. It is known that gastrointestinal allergies generally affect infants and children younger than two years of age [20]. On the other hand, children with juvenile polyps are known to present for review at a mean age of four years [25].

One of the main limitations of this multicenter study was its retrospective nature. We were unable to accurately report minor complications associated with colonoscopy in infants and young children due to differences in the criteria for recording complications among the three institutions. Determining these complications would have enabled us to enhance the usage profile for colonoscopy in infants and young children. Further follow-up including additional immunological or genetic testing may reveal more monogenic or other Mendelian forms of IBD.

## Conclusions

In the present study, we elucidated the role of colonoscopy in infants and young children. The most common diseases found by colonoscopy in this age group were EGIDs, IBD, and polyps/polyposis. The increasing trend of patients presenting with IBD and EGIDs worldwide means that the role of colonoscopy in infants and younger children will be more important in the future.

## Data Availability

The datasets generated and analyzed during the current study are available from the corresponding author on reasonable request.

## Abbreviations

CD: Crohn’s diseases
EGIDs: Eosinophilic gastrointestinal disorders (including gastrointestinal allergy)
IBD: Inflammatory bowel diseases
MPS: Mucosal prolapse syndrome
UC: Ulcerative colitis
